# miR-146a-5p impairs melanoma resistance to kinase inhibitors by targeting COX2 and regulating NFkB-mediated inflammatory mediators

**DOI:** 10.1186/s12964-020-00601-1

**Published:** 2020-09-23

**Authors:** Elisabetta Vergani, Matteo Dugo, Mara Cossa, Simona Frigerio, Lorenza Di Guardo, Gianfrancesco Gallino, Ilaria Mattavelli, Barbara Vergani, Luca Lalli, Elena Tamborini, Barbara Valeri, Chiara Gargiuli, Eriomina Shahaj, Marina Ferrarini, Elisabetta Ferrero, Macarena Gomez Lira, Veronica Huber, Michele Del Vecchio, Marialuisa Sensi, Biagio Eugenio Leone, Mario Santinami, Licia Rivoltini, Monica Rodolfo, Viviana Vallacchi

**Affiliations:** 1 Unit of Immunotherapy of Human Tumors, Fondazione IRCCS Istituto Nazionale dei Tumori, Via Venezian 1, 20133 Milan, Italy; 2 Platform of Integrated Biology, Department of Applied Research and Technological Development, Fondazione IRCCS Istituto Nazionale dei Tumori AmadeoLab, Milan, Italy; 3 Department of Pathology, Fondazione IRCCS Istituto Nazionale dei Tumori, Milan, Italy; 4 Unit of Oncology, Fondazione IRCCS Istituto Nazionale dei Tumori, Milan, Italy; 5 Melanoma and Sarcoma Surgery Unit, Fondazione IRCCS Istituto Nazionale dei Tumori, Milan, Italy; 6 Department of Medicine and Surgery, University of Milano-Bicocca, Monza, Italy; 7 Experimental Oncology, San Raffaele Scientific Institute, Milan, Italy; 8 Biology and Genetics, Department of Neurosciences Biomedicine and Movement Sciences, University of Verona, Verona, Italy

**Keywords:** Melanoma resistance, BRAF/MEK inhibitors, microRNA, miR-146a-5p, COX2

## Abstract

**Background:**

Targeted therapy with BRAF and MEK inhibitors has improved the survival of patients with BRAF-mutated metastatic melanoma, but most patients relapse upon the onset of drug resistance induced by mechanisms including genetic and epigenetic events. Among the epigenetic alterations, microRNA perturbation is associated with the development of kinase inhibitor resistance. Here, we identified and studied the role of miR-146a-5p dysregulation in melanoma drug resistance.

**Methods:**

The miR-146a-5p-regulated NFkB signaling network was identified in drug-resistant cell lines and melanoma tumor samples by expression profiling and knock-in and knock-out studies. A bioinformatic data analysis identified COX2 as a central gene regulated by miR-146a-5p and NFkB. The effects of miR-146a-5p/COX2 manipulation were studied in vitro in cell lines and with 3D cultures of treatment-resistant tumor explants from patients progressing during therapy.

**Results:**

miR-146a-5p expression was inversely correlated with drug sensitivity and COX2 expression and was reduced in BRAF and MEK inhibitor-resistant melanoma cells and tissues. Forced miR-146a-5p expression reduced COX2 activity and significantly increased drug sensitivity by hampering prosurvival NFkB signaling, leading to reduced proliferation and enhanced apoptosis. Similar effects were obtained by inhibiting COX2 by celecoxib, a clinically approved COX2 inhibitor.

**Conclusions:**

Deregulation of the miR-146a-5p/COX2 axis occurs in the development of melanoma resistance to targeted drugs in melanoma patients. This finding reveals novel targets for more effective combination treatment.

**Video Abstract**

**Graphical Abstract:**

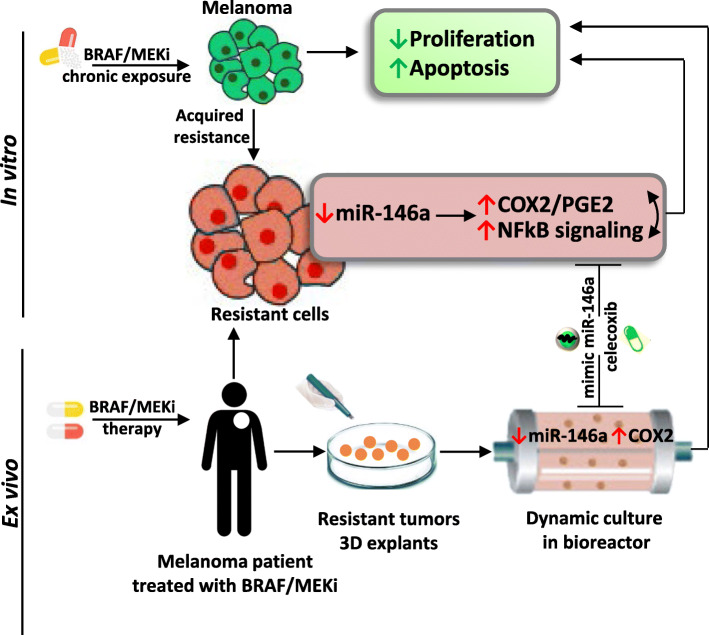

## Introduction

Treatment with BRAF and MEK inhibitors (BRAF/MEKi) has significantly improved the outcome of metastatic cutaneous melanoma patients with BRAF-mutated tumors. However, in the majority of patients, the clinical benefits are limited by the emergence of drug resistance [1]. Although BRAF/MEKi-resistant patients can benefit from different therapeutic opportunities, such as immune checkpoint blockade, strategies aimed at impairing the onset of resistance by drug combinations remain an urgent unmet clinical need.

The complex network of genomic modifications driving tumor evolution under pressure from drug treatment leads to MAPK signaling reactivation and/or AKT pathway sustainment and strongly conditions the tumor microenvironment (TME) [2]. Despite treatment, epigenetic changes governing drug-induced melanoma cell plasticity and disease progression activate protumorigenic signaling pathways that include microRNAs (miR) as regulators [3]. miR act in a cell context-dependent manner by playing key regulatory roles in cell survival and proliferation signaling pathways, contributing to the acquisition of BRAF/MEKi resistance [4, 5]. Our previous work highlighted the link between the acquisition of an HIF1/NFkB-directed proinflammatory phenotype and the dysregulation of a set of miR involved in prosurvival signaling pathways in BRAFi-resistant melanoma cell lines [6]. The acquisition of an HIF1/NFkB-directed proinflammatory phenotype was characterized by the induced production and release of several factors, including TNFα, CCL5, IL6, VEGFA, CXCL8, PGE2 and CCL2, which had a prosurvival effect on melanoma cells [6].

miR-146a-5p (miR-146a) is transcriptionally regulated by NFkB. It negatively controls NFkB-associated signaling pathways and release of proinflammatory cytokines through downregulating IRAK1 and TRAF6, and by negatively regulating expression of TNFα [7–11]. In melanoma, antithetic roles for miR-146a as both a tumor suppressor and tumor promotor were described [12–15], and its genetic polymorphism was associated with melanoma risk [16]. In particular, miR-146a overexpression was reported to promote melanoma cell growth by downregulating its direct targets NUMB and LFNG, which are involved in the NOTCH/PTEN/AKT pathway [12, 13]. Other investigators reported miR-146a involvement in the regulation of melanoma cell migration and invasive ability via targeting SMAD4 [15], ITGAV and ROCK1 [13]. Nonetheless, in other tumor types, such as breast, pancreatic and gastric cancer, miR-146a was reported to suppress cell proliferation, invasion and metastasis by repressing EGFR expression [7]. In lung cancer, miR-146a overexpression was shown to restore cisplatin sensitivity by targeting cyclin J and ATG12, while in liver cancer, miR-146a expression increased the radiosensitivity of tumor cells by downregulating RPA3 expression [7]. Of note, by negatively regulating EGFR expression, miR-146a could enhance the antitumor effects of drugs targeting this receptor [7].

Among the proinflammatory factors involved in tumor progression, cyclooxygenase-2 (COX2) and its product prostaglandin E2 (PGE2) have been found to be upregulated in many cancer types, favoring cell growth and survival and contributing to the generation of an immune suppressive TME [17]. Prostaglandin production depends on the activity of two isoforms of the COX enzyme, COX1 and COX2. While COX1 is constitutively expressed, COX2 is induced by different factors, such as growth factors and proinflammatory cytokines, including CCL2. Celecoxib is a nonsteroidal anti-inflammatory drug that specifically inhibits COX2, commonly used for the treatment of rheumatoid arthritis [18]. This drug is currently being repurposed in a clinical trial evaluating its treatment and prevention of cancer [18].

Notably, low miR-146a levels have been found in many tumor types, including melanoma [7], and were associated with increased COX2 levels in lung cancer [19]. However, the implication of the miR-146a/COX2 axis and its potential inhibition by celecoxib has not yet been investigated in the context of BRAF/MEKi resistance. Here, we identified the miR-146a/COX2 axis as playing a critical role in the mechanisms sustaining melanoma resistance; thus, this axis represents a druggable signaling pathway for improving the effects of BRAF/MEKi therapy in melanoma patients.

## Methods

### Cell cultures and reagents

The melanoma cell lines used in this study were described previously [6] and were periodically checked for mycoplasma and authentication by STR analysis (Gene Print 10 System, Promega, Fitchburg, WI, USA). Cell Counting kit 8 (CCK8, Sigma) was used to determine cell proliferation, LDH cytotoxicity assay kit (Pierce, Waltham, MA, USA) for LDH release, and Caspase-Glo 8 and 3/7 assays for detecting activated caspases (Promega, Fitchburg, WI, USA). The Prostaglandin E2 ELISA Kit (Cayman Chemical, Ann Arbor, MI, USA) was used for the measurement of PGE2 from culture supernatant of melanoma cells and of 3D cultures. Vemurafenib (PLX4032), Dabrafenib (BRAFi) and Trametinib (MEKi) were purchased (Selleck, Houston, TX, USA) and used at 3 μM for PLX4032 and 3 nM for BRAF/MEKi; recombinant sTRAIL (Vinci Biochem, Florence, Italy) was used at 50 ng/mL and the COX2 inhibitors celecoxib and NS398 (Sigma) at 50 μM.

For cells transfection of miR mimic, inhibitor and control oligos (50 nM), PTGS2 siRNA or siRNA control (100 nM) (Thermo Fisher Scientific, Waltham, MA, USA), Metafectene was used (Biontex, Munich, Germany). Transfection efficiency was monitored by qRT-PCR for miR-146a and by western blot for COX2 protein expression at 72 h. For Luciferase Assay, pLightSwitch-3’UTR-PTGS2 or the pLightSwitch empty vector (1 μg) (Active Motif, Carlsbad, CA, USA) were cotrasfected with either miR-146a mimic or negative control for 48 h and the LightSwitch Luciferase assay system (Active Motif) was used to analyze the luminescence production. All experiments were performed at 72 h after treatment, seeding 1,5 × 10^4^ cells/well in 96 wells plates.

### Western blot analysis and antibody array

Total protein was extracted from transfected cells using RIPA buffer (Thermo Fisher Scientific) and quantified using BCA protein assay (Bio-Rad, Hercules, CA, USA). Cell lysates (30 μg) were resolved on 4–12% SDS-PAGE and transferred to nitrocellulose membranes (Thermo Fisher Scientific); membranes were incubated with specific antibodies (supplementary Table S1). Peroxidase-conjugated secondary antibodies anti-mouse immunoglobulin and anti-rabbit immunoglobulin G were used. Antibody Array kit for Human Apoptosis Signaling (Raybiotech, Peachtree Corners, GA, USA) was used to test protein lysates. Chemiluminescence was measured by Uvitec Imaging System (Cleaver Scientific, Cambridge, UK) and quantified by Nine-Alliance software.

### RNA extraction and qRT-PCR analysis

RNA was extracted from melanoma cells and specimens with the NucleoSpin RNA isolation kit (Macherey Nagel, Bethlehem, PA, USA). Extracted RNAs were quantified spectrophotometrically, and the absorbance ratio at 260/280 and 260/230 were measured to assure RNA quality and purity. qRT-PCR analysis was performed using Thermo Fisher Scientific reagents for gene transcripts and Exiqon reagents for miR (supplementary Table S1). The endogenous controls used for normalization were ACTB and RPL13A for genes and U6 snRNA for miR. qRT-PCR was carried out in triplicate and run on the ABI Prism 7900HT or on the QuantStudio 7 Flex instruments and analysis was performed using SDS software, version 2.2.2 and with QuantStudio 6 and 7 Flex software. The results are presented as 2^−ΔCt^ ± SD for direct comparisons.

### miR and gene expression profiling data analysis

miR expression profiles were generated using NanoString nCounter Human v2 miR Expression Assay that contains 800 human endogenous miR. Raw data were normalized and log2-transformed using the NanoStringNorm R package [20], setting the required parameters to the following values: Probe.Correction.Factor to “adjust”; CodeCount to “sum”; Background to “mean”; SampleContent to “top.geo.mean” and OtherNorm to “none”. Raw and processed miR expression data are available at the NCBI Gene Expression Omnibus with accession date GSE141314. Raw miR expression data for GSE67635 [21] and GSE68841 [6] were downloaded from GEO and pre-processed using limma [22] with “normexp” method for background correction, quantile normalization and log2 transformation. Replicated probes were collapsed calculating the average expression and for each miR the probe with highest variance across samples was selected. Differential expression analysis was carried out using limma [22] (paired design for NanoString data). *P*-values were corrected for multiple testing using the Benjamini-Hochberg false discovery rate (FDR) method. A nominal *p*-value < 0.05 was applied to select differentially expressed miR for NanoString data and an FDR < 0.05 for GSE67635 and GSE68841.

Gene expression profiles of metastatic tumor samples were generated using HumanHT-12 WG-DASL V4.0 R2 expression beadchip (Illumina, San Diego, CA, USA). RNA labeling, processing and hybridization were performed according the manufacturer’s standard protocols. Microarrays were scanned with Illumina BeadArray Reader and raw expression data were obtained using Illumina BeadStudio v3.3.8 and processed using the lumi Bioconductor package [23] as previously described [6]. The data were deposited in GEO with accession number GSE141484. Functional analyses were generated through the use of Ingenuity pathway Analysis (IPA) (Qiagen, Hilden, Germany).

### 3D cultures of tumor explants in RCCS bioreactor

3D Cultures of Melanoma Tissue explants were set in Rotary Cell Culture System Bioreactor (RCCS) (Synthecon Inc., Houston TX, USA) as described by Ferrarini [24]. Tumor tissue cubes were obtained by a 3 mm biopsy puncher from tumor specimens obtained from the pathologist, and cultured in duplicate in the bioreactor chambers for 3 days, in culture medium or in the presence of BRAF/MEKi (3 nM) and/or miR-146a mimic or negative control synthethic oligos (50 nM). When recovered, samples were halved, a part was fixed in formalin and paraffin-embedded for histopathology, and a part was fixed in RNAlater solution (Thermo Fisher Scientific) for RNA analysis. Quantification of secreted cytokines in the 3D culture supernatant was carried by Cytometric Bead Array (CBA, BD Biosciences, San Jose, CA, USA) using the BD software FCAP Array v3.0.1.

### Immunohistochemistry

Melanoma sections were stained by specific antibodies (supplementary Table S1) for ki67, cleaved Caspase 3, phospho-ERK, and COX2 after antigen retrieval performed by heating in a pressure cooker with 0,5 mM EDTA pH 8 for 15 or 20 min and using a peroxidase-labelled polymer (UltraVision Quanto Detection System HRP Polymer, Thermo Fisher Scientific) and brown DAB as a chromogen (Dako Agilent, Glostrup, Denmark). Sections were scanned using the Aperio ScanScope XT systems (Aperio Technologies, Leica Microsystems).

### Statistical analysis

Statistical analyses were performed using Prism software v.5 and v.8 (La Jolla, CA, USA). Comparisons between continuous variables in two groups were performed using an unpaired two tailed Student’s t-test or Mann-Whitney U test. For comparisons involving more than two groups, one-way ANOVA was used, followed by Bonferroni correction. For comparisons between two dose-response curves, two-way ANOVA followed by Bonferroni correction was used. The correlation between linear variables was calculated using Pearson or Spearman’s correlation coefficients. Drug interaction was evaluated by a standard approach that allows a value to be assigned to a drug combination (interaction index); the interaction index was calculated as the ratio between expected cell growth and observed cell growth. If the experimentally measured effect of the drug combination was equal to, higher than or lower than 1, the combination was considered to be additive, synergistic or antagonistic, respectively. Data are presented as the mean ± SD.

## Results

### miR-146a is regulated in BRAFi-resistant melanoma cell lines

**Fig. 1:**
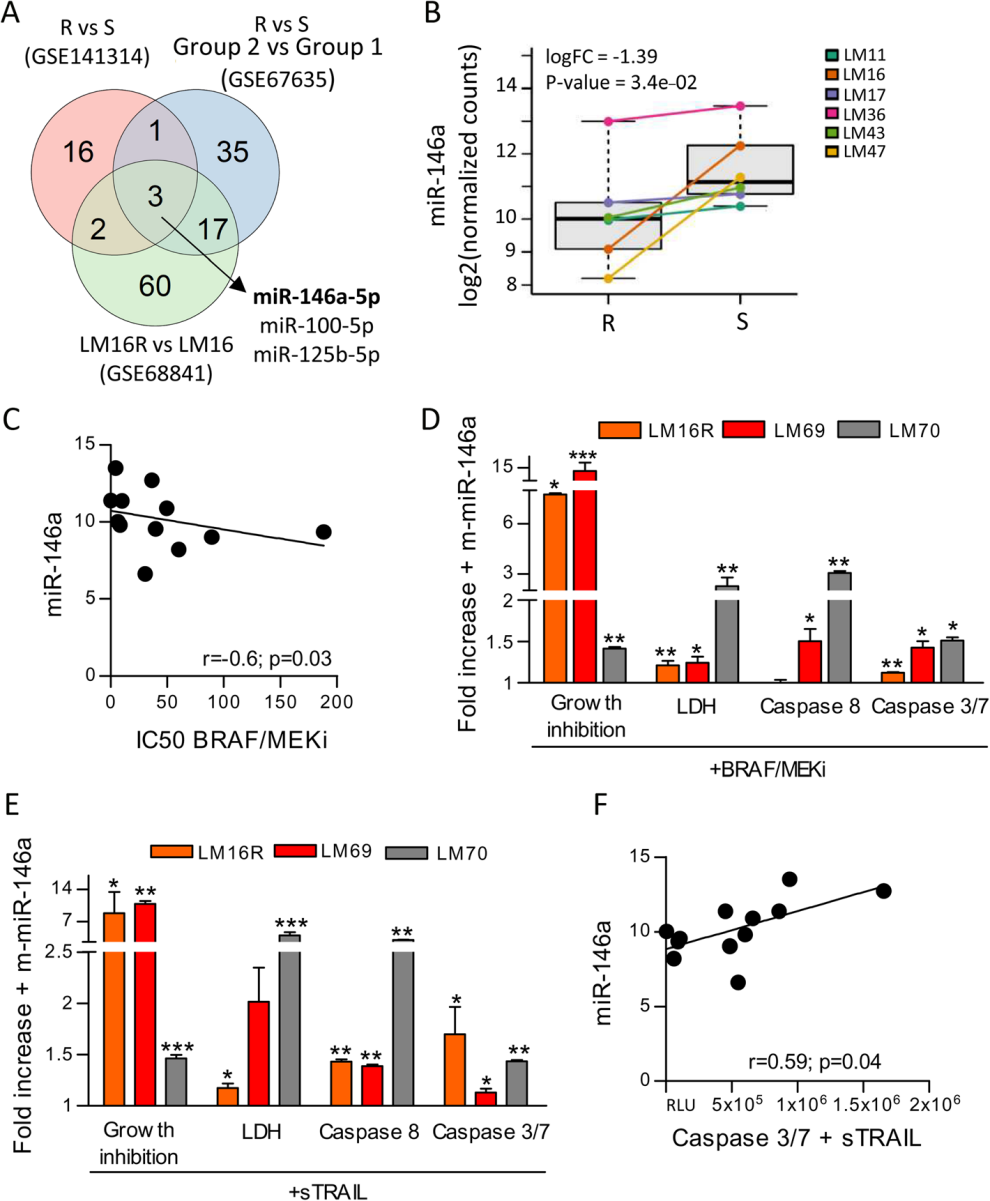
Downregulation of miR-146a is associated with resistance to BRAF/MEKi in melanoma cell lines and its manipulation affects drug response and TRAIL-induced apoptosis. **a** Venn diagram illustrating common miR associated with BRAF/MEKi resistance in GSE141314, GSE67635 and GSE68841 datasets. **b** miR-146a shows significant downregulation in melanoma resistant cells (R) compared with their sensitive counterparts (S). logFC: log_2_ Fold Change. *P*-value obtained from differential expression analysis performed with limma package. **c** Inverse correlation between miR-146a expression levels and IC50 values of combined BRAF/MEKi in melanoma cell lines (Spearman analysis). **d** Forced expression of miR-146a (+m-miR-146a) increases the effects of BRAF/MEKi treatment in LM16R, LM69 and LM70 cell lines, as shown by reduced cell growth and increased cell cytotoxicity and apoptosis, evaluated by CCK8, LDH and caspase 8 and 3/7 activity. LM69 and LM70 short term melanoma cultures were generated from treatment resistant melanoma lesions surgically excised from two patients. Based on qRT-PCR results, upon transfection miR-146a levels were up to 70-fold higher (range 76–1223) than the levels detectable in cells transfected with control oligos. Data are plotted as fold increase compared to cells transfected with mimic negative control. _*_: *p* < 0.05, _**_: *p* < 0.01, _***_: *p* < 0.0001 by Student’s unpaired t test. **e** Forced expression of miR-146a increased the effect of treatment with sTRAIL. **f** Positive correlation between the levels of miR-146a expression and of activated caspase 3/7 induced by sTRAIL treatment (Spearman analysis). RLU: Relative Light Unit. Results shown are representative of 2 experiments performed in triplicate (**d, e**)

To expand the analysis of the miR involved in establishing a resistant phenotype, we analyzed the miR expression profiles of six melanoma cell lines which developed acquired resistance to vemurafenib (PLX4032) and cross resistance to dabrafenib (BRAFi), trametinib (MEKi) and the combination of dabrafenib and trametinib (BRAF/MEKi) and their parental drug-sensitive counterparts (supplementary Fig. S1A). We identified 22 differentially expressed miR between the resistant and sensitive cells; of these miR, 6 were upregulated, and 16 were downregulated in the resistant cells (supplementary Table S2). To strengthen our findings and focus on miR which could be relevant for resistance, acquired and intrinsic, we intersected these 22 miR to those previously identified in our laboratory in a similar profiling setting (GSE68841; 6) and to those differentially expressed between melanoma cell lines sensitive (cluster 1) or intrinsically resistant to the BRAFi PLX4720 (cluster 2) (GSE67635, 21). Three miR were commonly modulated in all three datasets (Fig. 1a). Two of them, miR-100-5p and miR-125b-5p, upregulated in resistant cells, have been previously investigated [6]. The third miR, miR-146a, was downregulated in resistant cells and its down-regulation was indeed confirmed in each of the six BRAFi-resistant melanoma cell lines of the current study compared to their sensitive counterparts (Fig. 1b). First evidence of its association with drug resistance was given by the inverse correlation of miR-146a and the IC50 values of BRAF/MEKi used in combination or as single agents (Fig. 1c and supplementary Fig. S1B).

### Modulation of miR-146a affects the BRAF/MEKi drug response in melanoma cells

To assess whether miR-146a expression levels affect the drug sensitivity of melanoma cells, we transiently manipulated its expression in gain- and loss-of-function assays and tested the effects on kinase inhibitor treatment. miR-146a hyperexpression in cell lines with acquired or intrinsic resistance significantly reduced cell growth, increased cytotoxicity and apoptosis induced by drug treatment compared with control conditions (Fig. 1d and supplementary Fig. S1CE). Conversely, following miR-146a inhibitor transfection, the effect of drug treatment on cell cytotoxicity and apoptosis was reduced in sensitive LM16 cells (supplementary Fig. S1D).

Interestingly, we observed that resistance to BRAF/MEKi was extended to apoptosis determined by exposure to recombinant soluble TRAIL (sTRAIL), a functional mediator of cytotoxic immune cells (supplementary Fig. S1A). miR-146a overexpression in drug-resistant cells increased their sensitivity to sTRAIL-induced apoptosis (Fig. 1e, supplementary Fig. S1E), while miR-146a inhibition was protective (supplementary Fig. S1D). miR-146a expression levels were negatively correlated with sTRAIL-induced caspase 3/7 levels (Fig. 1f), further supporting the functional contribution of miR-146a to regulating cell apoptosis.

### Forced expression of miR-146a reduces drug resistance via AKT/ERK and NFkB signaling cascade

**Fig. 2:**
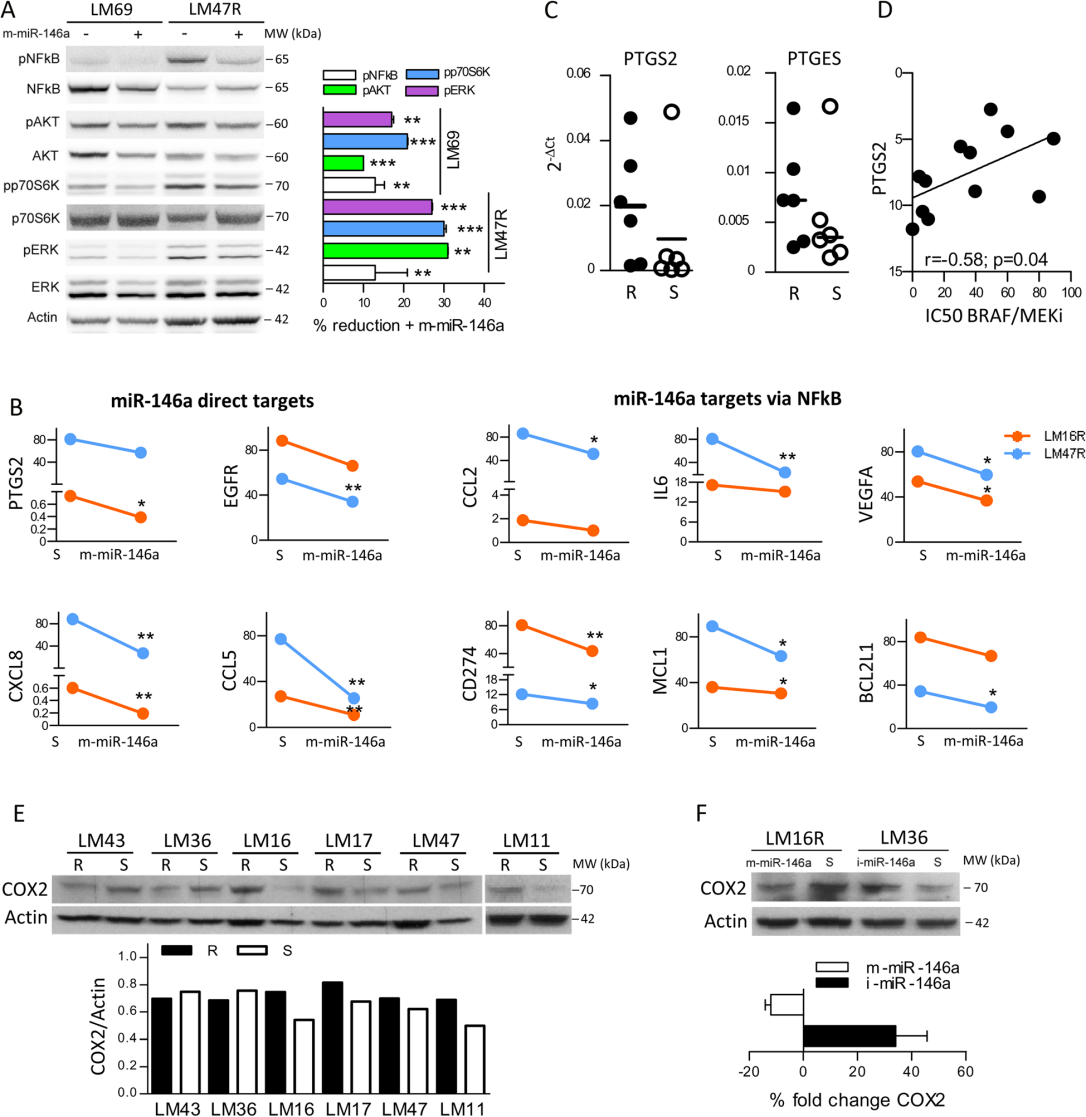
miR-146a regulates NFkB-related signaling pathways associated with resistance and directly targets PTGS2. **a** Immunoblot showing reduced levels of NFkB, AKT, p70S6K, and ERK in melanoma cells transfected with synthetic miR-146a (m-miR-146a) compared to mimic negative control transfectants (left). Reduction of phosphorylated proteins in miR-146a transfectants calculated by signal quantification (right). **b** Reduced expression of miR-146a target genes in transfectants (m-miR-146a: miR-146a mimic; S: mimic scrambled control). **c** Expression levels of PTGS2 and PTGES genes in resistant cell lines (R) compared to their sensitive counterparts (S). **d** Positive correlation between PTGS2 expression and IC50 values of BRAF/MEKi in melanoma cell lines (Spearman analysis). **e** Higher levels of COX2 protein expression in resistant cells (R) compared to their sensitive counterparts (S). Specific signals were quantified and expressed as the ratio of COX2/actin intensity. **f** Reduction of COX2 protein expression in LM16R resistant cells transfected with miR-146a mimic (m-miR-146a) and increased levels in LM36 parental sensitive cells transfected with miR-146a inhibitor (i-miR-146a). Changes in COX2 expression were calculated by signal quantification. *P* values were calculated by Student’s unpaired t test in A and F, and by Mann-Whitney U test in B. _*_: *p* < 0.05, _**_: *p* < 0.01, _***_: *p <* 0.0001

BRAFi resistance is associated with a proinflammatory phenotype, characterized by the endogenous upregulation of the HIF1/NFkB-directed gene expression of cyto-chemokines, growth factors and other inflammatory mediators [6]. Because miR-146a is subject to NFkB regulation [8], we tested its involvement in drug resistance-induced NFkB modulation. miR-146a ectopic expression decreased NFkB activity (Fig. 2a), acting as a negative feedback loop regulator [9]. As expected, the prosurvival AKT/mTOR/ERK pathway and MAPK signaling pathway were downregulated, as indicated by the reduced pAKT, AKT, pp70S6K and pERK levels (Fig. 2a). The link between miR-146a downregulation and melanoma cell growth is sustained by the results of IPA analysis showing that putative miR-146a target genes, upregulated in GSE68841 [6], associate to “Cellular Growth and Proliferation” and to “Cellular Movement” Network functions.

To identify genes regulated by miR-146a via direct targeting or NFkB, we first extracted those differentially expressed in the GSE68841 dataset [6] from the list of 1667 NFkB-transcribed genes reported in the database built by Yang [25]. Twenty-four out of 527 common genes were validated direct target genes of miR-146a according to the miRWalk 2.0 database (supplementary Table S3) [26]. They included CCL5, CXCL8, EGFR and PTGS2 genes related to the proinflammatory and invasive signatures of resistant cells [6, 27]. In addition, among the genes indirectly regulated by miR-146a through NFkB activity, we found CCL2, IL6, and VEGFA, which contribute to the proinflammatory phenotype, CD274 (PDL1), which promotes melanoma cell proliferation [28], and BCL2L1 and MCL1 antiapoptotic genes. All these genes displayed reduced expression upon forced miR-146a expression in melanoma cells (Fig. 2b).

**miR-146a impairs drug resistance by regulating the expression of PTGS2 gene encoding COX2.**

Among the predicted miR-146a gene targets, the PTGS2 gene encoding COX2 attracted our attention because COX2 overexpression was reported to be associated with tumor progression and poor prognosis in melanoma [29–31]. COX2 transcript and protein expression levels were higher in several cells with acquired resistance compared to their sensitive counterparts and positively correlated with BRAF/MEKi resistance, revealing a direct association between COX2 and resistance (Fig. 2cde and supplementary Fig. S2B). Resistant cell lines also showed upregulation of PTGES, the gene encoding microsomal prostaglandin E2 synthase 1 (mPGES1), which is functionally coupled with COX2 in PGE2 production (Fig. 2c).

By luciferase reporter assays, we detected a significant decrease in luciferase activity upon melanoma cells transfection with the pLightSwitch-3’UTR-PTGS2 vector together with the miR-146a mimic when compared with controls, confirming a role for miR-146a in the post-transcriptional regulation of PTGS2 (supplementary Fig. S2A). Finally, forced miR-146a expression in resistant cells resulted in a decrease in COX2 protein levels, while inhibition of miR-146a in sensitive cells induced an increase in COX2 levels (Fig. 2f). These findings further confirm the involvement of miR-146a in COX2 regulation.

### Targeting COX2 restores melanoma sensitivity to BRAF/MEKi

**Fig. 3:**
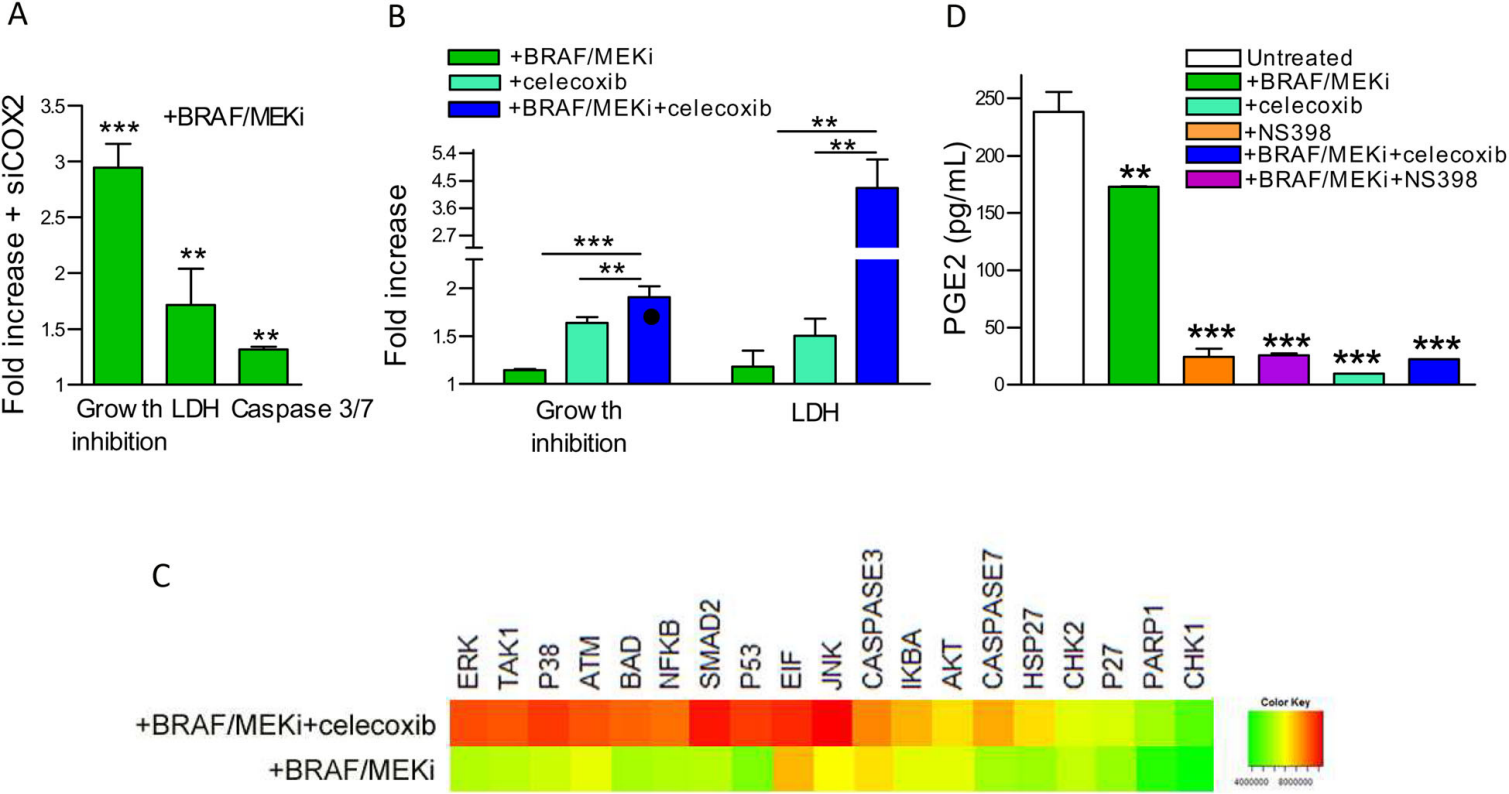
miR-146a spoils resistance of melanoma cells by repressing COX2 expression. **a** Silencing of COX2 by siRNA transfection (siCOX2) enhanced cell growth inhibition and increased cell cytotoxicity and apoptosis upon treatment with BRAF/MEKi. **b** Combined treatment with celecoxib increased cell growth inhibition and cytotoxicity by BRAF/MEKi. ●: interaction index = 1. **c** Increased apoptotic signaling after combination treatment with celecoxib and BRAF/MEKi. Phosphorylated and cleaved apoptotic factors showing increased expression in cells treated with the drug combination compared to BRAF/MEKi treated cells (Fold change > 1.3) by apoptosis signaling antibody array analysis. **d** Reduction of PGE2 release in culture media by treatment with the COX2 inhibitors celecoxib and NS398, alone or in combination with BRAF/MEKi. All experiments were carried with the LM47R cell line. *P* values were calculated by Student’s unpaired t test in B, and by one-way ANOVA followed by Bonferroni correction in D. _*_: *p* < 0.05, _**_: *p* < 0.01, _***_: *p* < 0.0001

The above results prompted us to investigate whether targeting COX2 may impact BRAF/MEKi resistance. COX2 knockdown by small interfering RNA (siCOX2) reduced COX2 expression levels and enhanced melanoma cell sensitivity to BRAF/MEKi (Fig. 3a and supplementary Fig. S2CD). Pharmacologic inhibition of COX2 activity by the selective inhibitor celecoxib and BRAF/MEKi significantly reduced cell growth and increased cell cytotoxicity (Fig. 3b and supplementary Fig. S2F). The combined treatment resulted in an additive inhibition of cell growth (interaction index = 1), a result also confirmed by dose-response assays (supplementary Fig. S2FG).

A screening of apoptosis-related proteins by antibody arrays showed that combined treatment significantly increased the phosphorylation of the proapoptotic proteins BAD and caspase 7 through activating SMAD2, JNK and p38/MAPK apoptosis-related signaling pathways. Moreover, induction of ATM signaling resulted in non canonical NFkB activation [32], determining the phosphorylation of components of the pathways involved in DNA damage-induced apoptosis, such as PARP1, p53 and TAK1 (Fig. 3c).

One consequence of COX2 overexpression is increased PGE2 production, which has multiple protumoral effects, including apoptosis resistance [17]. According to the high expression levels of COX2 and PTGES, resistant cell lines indeed secreted high levels of PGE2, which were significantly inhibited upon cell exposure to the COX2 inhibitors celecoxib and NS398, independent of BRAF/MEKi treatment (Fig. 3d and supplementary Fig. S2E). Taken together, these data indicate that the enhanced apoptotic effects induced by BRAF/MEKi in the presence of COX2 inhibition result from diminished PGE2 production, which reduces prosurvival signaling and activates apoptotic pathways and DNA damage signal transducers.

### BRAF/MEKi resistance affects the miR-146a/COX2 axis in melanoma patients

**Fig. 4:**
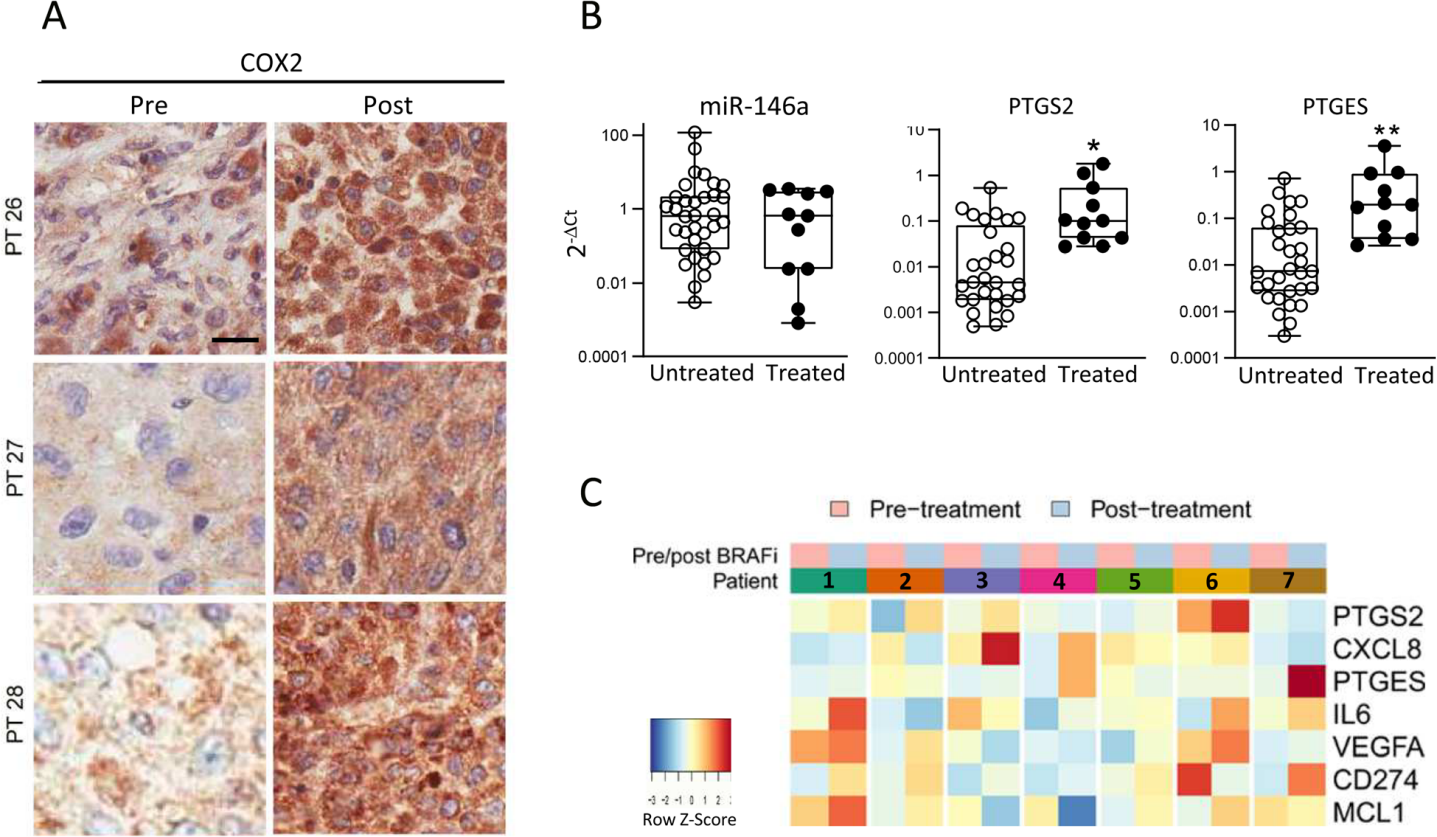
Treatment resistant tumors display miR-146a/COX2 axis deregulation. **a** COX2 immunostaining in tumors excised from patients progressing during BRAF/MEKi therapy (Post) compared to pre-therapy matched melanoma lesions (Pre). Scale bar: 10 μm. **b** Lower miR-146a and higher PTGS2 and PTGES expression levels in tumors progressing in patients during BRAF/MEKi therapy (Treated, *n* = 12) compared to tumors from unmatched untreated patients (Untreated, *n* = 31). _*_: *p <* 0.05, _**_: *p <* 0.01 by Mann-Whitney U test. **c** Heatmap showing the gene expression pattern of PTGS2, CXCL8, PTGES, IL6, VEGFA, CD274, MCL1 in progressing tumors (Post-treatment) compared to matched pre-therapy lesions (Pre-treatment) from seven metastatic melanoma patients receiving BRAFi therapy

To determine whether the miR-146a downregulation and COX2 upregulation observed in BRAF/MEKi-resistant melanoma cell lines in vitro also occurred in vivo in melanoma patients, we analyzed tumor samples surgically excised for local treatment from patients undergoing BRAF/MEKi therapy. Tissue immunostaining confirmed higher COX2 expression in tumor lesions progressing during BRAF/MEKi therapy compared to matched pretreatment lesions in 3 out of 7 tested cases (Fig. 4a and supplementary Table S4). High PTGS2 and PTGES and low miR-146a expression levels were detected in treatment-resistant tumors when compared to tumors from untreated patients (Fig. 4b). Despite the different numbers of treated and untreated samples analyzed and the diverse levels of gene expression detected in the tumor samples, significantly higher PTGS2 and PTGES was evident in tumors from treated patients. For miR-146a, although several untreated tumors show high expression levels, while on the contrary low levels are detected in treated specimens, no significant differences can be observed between untreated and treated samples.

Similar to what was observed in cell lines, miR-146a expression levels were inversely correlated with the levels of its target genes EGFR, CCL2 and BCL2L1 in melanoma metastases (supplementary Fig. S3). We then analyzed an in-house gene profiling dataset of seven matched metastatic tumor samples obtained from a cohort of patients before therapy and after the onset of treatment resistance for expression values of PTGS2 and of a number of additional direct or indirect miR-146a targets. In comparison to the matched pre-therapy sample, resistant lesions from five of the patients (Pts 1, 2, 3, 5 and 6) coordinately displayed higher expression of PTGS2. The remaining two patients (Pts 4 and 7) had instead lower expression of PTGS2 in their resistant sample. Different numbers of additional direct or indirect miR-146a targets, five in Pt 1, four in Pts 5 and 6, three in Pt 2 and two in Pt 3, displayed an expression trend similar to PTGS2. Among them, VEGFA, CD274, MCL1 were upregulated in most of the resistant lesions, whereas PTGES increase was never observed (Fig. 4c). Although an heterogenous pattern in term of gene expression levels was clearly displayed among patients, deregulation of the miR-146a/COX2 axis occurs in a subset of melanoma patients and is associated with the development of BRAF/MEKi drug resistance.

### Functional relevance of miR-146a/COX2 axis manipulation in BRAF/MEKi resistance

**Fig. 5:**
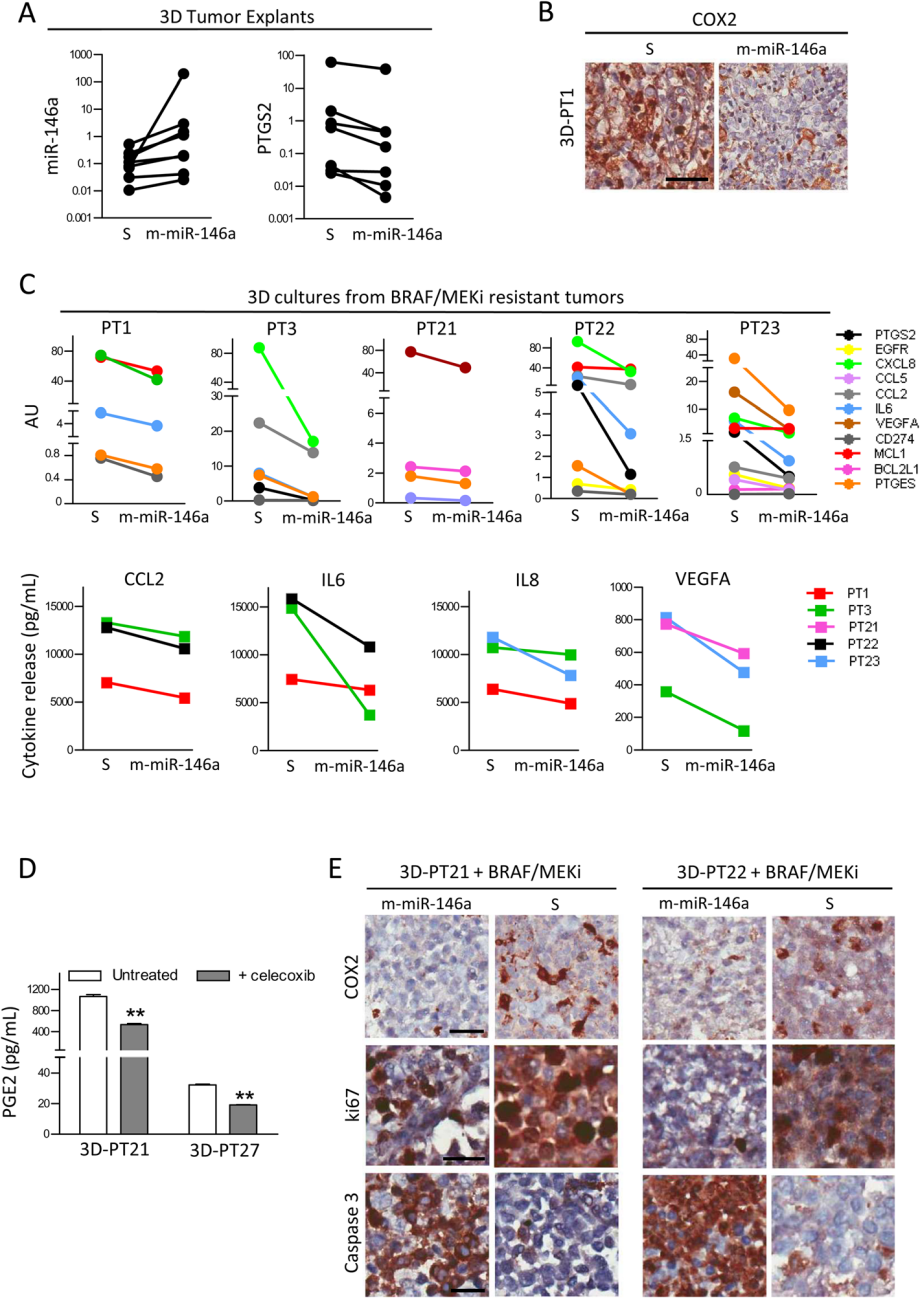
miR-146a overexpression reduces COX2 and increases drug sensitivity in 3D cultures from BRAF/MEKi-resistant tumors. **a** Overexpression of miR-146a upon transfection of specific mimic (m-miR-146a) in 3D tumor explants (left) downregulates COX2 expression compared to scrambled-transfected control (S) (right). **b** Reduced COX2 immunostaining in 3D cultures upon miR-146a forced expression (m-miR-146a) compared to mimic scrambled control (S). **c** Regulation of PTGS2 and miR-146a target genes (upper panel), and decreased release of CCL2, IL6, IL8 and VEGFA (lower panel) upon treatment with miR-146a mimic (m-miR-146a) and BRAF/MEKi in 3D cultures of resistant tumors in comparison to scrambled-transfected control (S). AU: arbitrary units. **d** PGE2 release in culture media from 3D tumor explants upon celecoxib treatment. _**_: *p <* 0.01 by Student’s unpaired t test. **e** Effects of treatment with BRAF/MEKi combined with transfection of miR-146a mimic (m-miR-146a) or of scrambled control (S) in resistant tumors: reduction of COX2 and of Ki67 positive cells and increase of cleaved caspase 3 immunostaining. Scale bars: 10 μm

To investigate the effects of miR-146a overexpression and COX2 inhibition on melanoma tumors, we performed 3D experiments with tumor explants using a short-term dynamic culture system in a bioreactor [24]. The preservation of TME architecture and cellularity by this culture system was confirmed in preliminary experiments (supplementary Fig. S4). Compared to control conditions, forced miR-146a expression led to increased miR-146a levels accompanied by decreased PTGS2 transcription and downregulation of most of the studied direct and indirect miR-146a target genes (Fig. 5ac and supplementary Fig. S5A).

In a set of 3D cultures from the lesions of 5 treatment-resistant metastatic patients, forced miR-146a upregulation led to reduced COX2 protein and gene levels and decreased PTGES, EGFR, CXCL8, CCL5, CCL2, IL6 and VEGFA, CD274, MCL1 and BCL2L1 levels (Fig. 5bc). These results indicated that downregulated miR-146a expression occurring through the acquisition of resistance to BRAF/MEKi-activated tumor-derived components contributes to melanoma survival and the generation of a tumor-promoting immunosuppressive TME; this finding was also confirmed by the reduced CCL2, IL6, IL8 and VEGFA secretion in the culture supernatants (Fig. 5c). Moreover, these observations led us to test the relevance of COX2 inhibition on PGE2 production in 3D tumor explants from a BRAF/MEKi-resistant patient and a treatment naïve patient. In both tumors, the COX2 inhibitor celecoxib significantly reduced PGE2 release, demonstrating that COX2 activity regulates PGE2 production also in tumor tissue (Fig. 5d). Consistently, a tissue analysis revealed that forced miR-146a expression remarkably reduced tumor cell proliferation in 3D cultured explants upon BRAF/MEKi treatment. These effects were observed in 3D cultures of BRAF-mutated melanoma tumors from naïve patients (supplementary Fig. S5B) and confirmed in tumors from patients undergoing treatment (Fig. 5e).

Our results strongly support a central role for the miR-146a/COX2 axis in modulating key components of tumor growth and survival associated with the onset of BRAF/MEKi resistance.

## Discussion

In the present study, we depict for the first time a role of miR-146a in controlling BRAF/MEKi resistance in melanoma cells, through a mechanism involving COX2. Chronic exposure to BRAFi induces miR-146a decrease in melanoma cells and increases expression of COX2, which boosts tumor cell survival to BRAFi resistance. Interestingly, we found that the increased BRAF/MEKi sensitivity induced by forced miR-146a expression in BRAF/MEKi-resistant cells were achieved by regulating COX2 expression. Our findings are consistent with previous observations demonstrating an inverse relationship between miR-146a and COX2 in lung cancer cells [19]. The analysis of tumors progressing in patients during BRAF/MEKi treatment revealed that miR-146a downregulation and the associated COX2 upregulation are not unique to in vitro models of BRAF/MEKi resistance as also occur in tumors in vivo. Due to the multiplicity of molecular alterations that characterize resistant melanoma, these results point to an important role of the miR-146a/COX2 axis in resistance. Moreover, the inhibition of COX2 by forced miR-146a expression in drug-resistant melanoma lesions is associated with the downregulation of immunosuppressive cyto-chemokines contributing to melanoma survival.

Decreased miR-146a expression was previously associated with drug resistance in lung, cervical, and hepatocellular cancer cells, and its overexpression suppressed cell growth and migration and improved drug sensitivity by inducing apoptosis [7]. Consistent with these findings, our study shows that miR-146a is significantly downregulated in six melanoma cell lines with acquired resistance and poorly expressed in two lines derived from drug-resistant tumors, and that its overexpression can restore sensitivity to BRAF/MEKi treatment by affecting both cell proliferation and apoptosis although with a different effect extent in the studied cell lines. Several studies have shown that decreased miR-146a expression leads to the overexpression of NFkB-mediated inflammatory factors contributing to immunosuppression and disease progression, and that miR-146a replacement can prevent the inflammatory state [8, 33]. Activation of NFkB signaling, along with low expression and activity of MITF, represent hallmarks of melanoma resistance to targeted therapy, predictive of poor prognosis for patients treated with BRAF/MEKi. NFkB-high transcriptional state could be present prior to therapy or could be induced by the acquisition of drug resistance. TNFα is a strong NFkB agonist, able to induce transcriptional changes promoting drug resistance [34]. In this view, we speculate that the downregulation of miR-146a could be considered an epigenetic mechanism contributing to the maintenance of the NFkB-high transcriptional state associated to resistance, because of its negative regulation of NFkB signaling and TNFα expression. In line with these findings, our experiments demonstrated that miR-146a ectopic expression in resistant cells modulated the levels of NFkB activity, downregulated the AKT/mTOR/ERK and MAPK signaling pathways, and reduced the expression of NFkB-induced mediators involved in cell growth and survival, including COX2 and EGFR, the immunosuppressive molecules IL8, CCL5, CCL2, IL6, VEGFA and PDL1, and the antiapoptotic genes BCL2L1 and MCL1.

miR-146a is involved in controlling the inflammatory response of innate immune system cells, particularly monocytes/macrophages [35]. Several studies have reported that miR-146a is associated with the negative regulation of immune activation and cancer-related immunosuppression [36]. For instance, miR-146a knock-out mice with melanoma survived longer [37], and miR-146a favors immunosuppression by increasing the regulatory T cell population in colorectal cancer [38]. In addition, miR-146a, one of the most studied myeloid miR [39], has been reported to serve as negative feedback modulator in the TLR4-mediated activation of NFkB–related genes [8] and promote M2 polarization in both humans and mice [40]. As we also reported, high miR-146a levels were associated with the induction of myeloid suppressor cells (MDSCs) and resistance to immunotherapy in melanoma patients [41]. Notably, miR-146a upregulation with concomitant increased COX2, PDL1, VEGFA, CCL2, IL6, IL8 and MCL1 expression characterized the MDSCs induced in vitro by melanoma extracellular vesicles from monocytes from healthy donors, suggesting cell type-specific regulation of the miR-146a/COX2 axis.

COX2 is an inducible enzyme essential in the biosynthesis of PGE2 through the enzyme mPGES1. Overexpression of COX2 is reportedly associated with a dismal prognosis in several tumor types [29, 30, 42]. Constitutive COX2 expression is triggered by pathways activated by oncogenic stimuli and cyto-chemokines, such as MAPK, PI3K/AKT and NFkB that are frequently hyperexpressed in most of resistant tumors [17]. Upregulation of the COX2/PGE2 axis favors proliferation, angiogenesis, invasion, apoptosis resistance, and the activation of immunosuppressive cells contributing to tumor progression and therapy resistance [43]. We showed that COX2 and PTGES are both overexpressed in several BRAF/MEKi-resistant melanoma cells and tumor tissues, and that COX2 knockdown significantly enhanced drug effects in melanoma cell. The COX2 selective inhibitor celecoxib alone and in combination with BRAF/MEKi has shown tumor inhibitory effects in preclinical melanoma studies [44, 45]. Clinical trials have also reported the effect of COX2 inhibitors, alone or combined with other treatment modalities, in cancer patients [17, 18]. However, there are no clinical reports investigating the treatment effect of COX2 inhibitors on BRAF/MEKi melanoma resistance. Moreover, it has been reported that COX2 can modulate PDL1 expression, and that celecoxib reduces PDL1 tumor expression in vitro and favors cytotoxic T cell responses [46, 47]. Other studies have described that COX2/mPGES1/PGE2 signaling regulates PDL1 and IDO in tumor-associated macrophages and MDSC cells, thus inducing an immunosuppressive phenotype [48–50]. In line with these reports, our data showed a coordinated expression of COX2 and PDL1 in resistant melanoma lesions and in melanoma cells upon forced miR-146a expression.

Our data show that celecoxib treatment reduced PGE2 production in resistant cells and in 3D cultures of resistant tumors, and its combination with BRAF/MEKi increased the drugs’ effect. Celecoxib-induced cell death is characterized by the activation of intrinsic and extrinsic apoptosis pathways and of NFkB signaling cascades induced by DNA damage, in line with published literature [18, 32, 51]. Our results support the targeting of COX2 by direct inhibition with selective drugs or by miR-146a delivery represents an option to improve the response to targeted therapy in melanoma patients.

## Conclusion

**Fig. 6:**
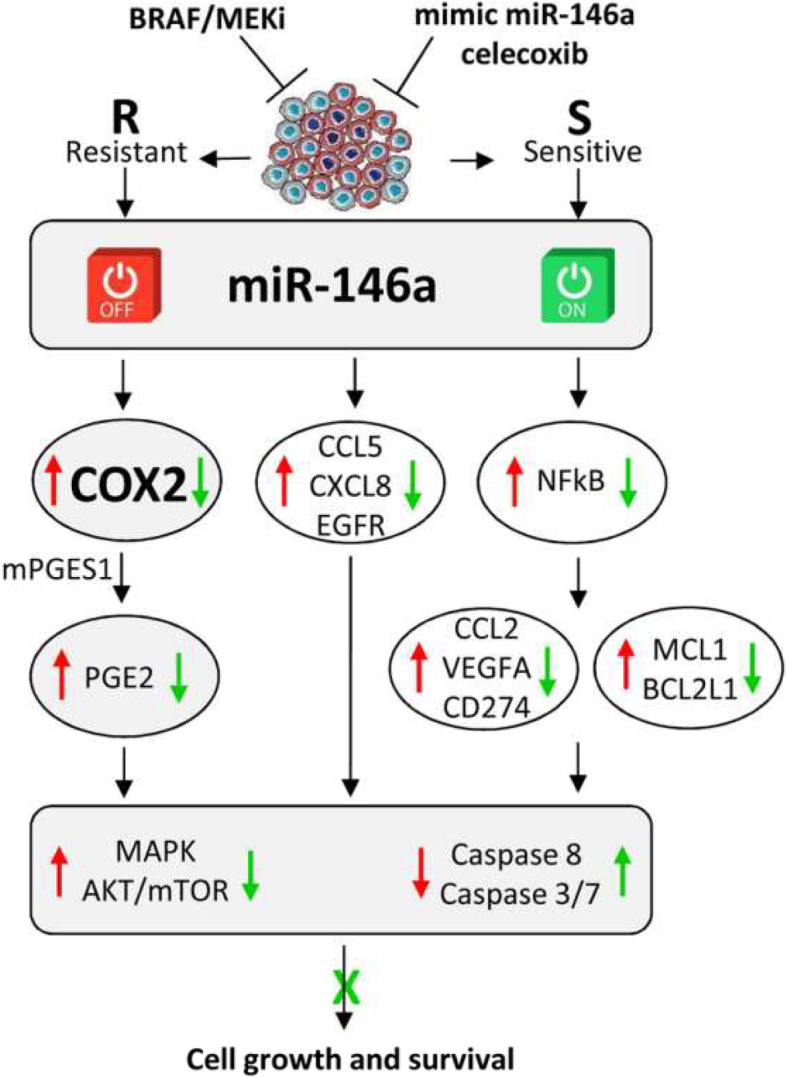
Schematic representation of the miR-146a/COX2 axis associated with BRAF/MEKi resistance

We here report a tumor-suppressive role of miR-146a in BRAF/MEKi-resistant melanoma and demonstrate that the manipulation of miR-146a/COX2/PGE2 axis can restore BRAF/MEKi chemosensitivity (Fig. 6).

## Supplementary information

**Additional file 1: Supplementary Table S1.** List of used antibodies and molecular probes. **Supplementary Table S2.** Differentially expressed miR between cell lines with acquired resistance to vemurafenib and their sensitive counterparts. **Supplementary Table S3.** Genes directly targeted by miR-146a among the NFkB-regulated genes differentially expressed in LM16R/LM16 dataset (GSE68841). **Supplementary Table S4.** Characteristics of patients treated with vemurafenib and their matched pre- and post-treatment melanoma metastasis (GSE141484).

**Additional file 2: Supplementary Fig. S1.** Altered miR-146a expression influences BRAF/MEKi sensitivity and apoptosis in melanoma. A) Boxplots representing IC50 values of 6 matched PLX4032-resistant (R) and sensitive (S) melanoma cell lines to PLX4032 (vemurafenib), BRAFi (dabrafenib), MEKi (trametinib), to the combined treatment BRAF/MEKi, and to sTRAIL-induced apoptosis. **B**) Inverse correlation between miR-146a expression levels and IC50 values of PLX4032, BRAFi and MEKi in melanoma cell lines (Spearman analysis). **C**) Forced expression of miR-146a (+m-miR-146a) in LM16R cell line and in LM69 and LM70 short term cultures increased the effects of PLX4032 treatment as shown by reduced cell growth and increased cell cytotoxicity and apoptosis, evaluated by CCK8, LDH and caspase 8 and 3/7 activity. **D**) Inhibition of miR-146a expression (i-miR-146a) in LM16 cells increased cell proliferation and decreased the release of LDH and the apoptosis rate as evaluated by CCK8, LDH and caspase 3/7 activity. **E**) Overexpression of miR-146a (m-miR-146a) upon PLX4032, BRAF/MEKi and sTRAIL treatments in LM47R cells increased cell cytotoxicity and apoptosis, as evaluated by LDH and caspase 8 and 3/7 activity. Data are plotted compared to scrambled control. _*_: *p* < 0.05, _**_: *p* < 0.01, _***_: *p* < 0.0001 by Student’s unpaired t test. **Supplementary Fig. S2.** COX2 inhibition increases sensitivity to PLX4032 and to BRAF/MEKi and reduces PGE2 release. **A**) Relative luciferase activity after co-transfection of PTGS2 3’UTR luciferase reporter vector or control vector with miR-146a mimic or mimic negative control. Experiment was performed in LM16R cells. **B**) Inverse correlation between miR-146a expression levels and IC50 values of PLX4032 in melanoma cell lines (Spearman analysis). **C**) Western blot analyses showing downregulation of COX2 after transfection with siRNA against COX2 (siCOX2) compared to scrambled control (S). COX2 protein levels were downregulated to 15%, as determined by quantification of the signal and expressed as the ratio of COX2/Actin intensity. Experiment was performed with LM47R cells. **D)** Silencing of COX2 by siRNA transfection enhanced cell growth inhibition and increased cell cytotoxicity and apoptosis upon treatment with PLX4032 compared to scrambled control. **E**) PGE2 release in culture supernatants following 24 h treatment with the COX2 inhibitors celecoxib and NS398, alone or in combination with BRAF/MEKi. **F)** Combined treatment with celecoxib increased cell growth inhibition and cytotoxicity by PLX4032. ●: interaction index = 1. **G)** Dose-dependent effect of celecoxib alone (5, 10, 20, 40, 50 and 80 μM) and combined with PLX4032 (3 μM) on cell growth. ●: interaction index = 1 at the dose of 50 μM of celecoxib combined with PLX4032. *P* values were calculated by Student’s unpaired t test in A, D and F, by one-way ANOVA followed by Bonferroni correction in E, and by two-way ANOVA followed by Bonferroni correction in G. _*_: *p* < 0.05, _**_: *p* < 0.01, _***_: *p* < 0.0001. **Supplementary Fig. S3.** miR-146a expression levels are inversely correlated with levels of target genes. Analysis of correlation between miR-146a expression and its target genes EGFR, CCL2 and BCL2L1 in metastatic melanoma specimens. The rp and *p* values resulting from Pearson analysis are shown. **Supplementary Fig. S4.** Dynamic culture in bioreactor preserves TME architecture of 3D tumor explants. Representative histological images of H&E-stained 3D tumor sections after 3 days culture in bioreactor. The marked areas in the left panels are shown at higher magnification in the right panels to display the preserved cellularity and tumor tissue histo-architecture. Scale bar 80 μM. **Supplementary Fig. S5.** miR-146a overexpression increases drug sensitivity in 3D cultures of tumor explants. **A**) Modulation of direct and indirect miR-146a target genes upon transfection of miR-146a mimic (m-miR-146a) compared to scrambled-transfected control (S) in 3D cultures of BRAF-mutated melanoma tumors from naïve patients. AU: arbitrary units. **B**) Decreased staining for Ki67 and pERK proliferating cells and increased Caspase 3 positive cells in 3D cultures upon treatment with BRAF/MEKi and transfection with miR-146a (m-miR-146a) or scrambled control (S). Scale bar 10 um.

## Data Availability

All data generated or analyzed during this study are included either in this article or in the supplementary information files.

## Abbreviations

ACTB: Actin Beta
BRAF/MEKi: BRAF inhibitor, MEK inhibitor
CCK8: Cell Counting Kit 8
CCL2: C-C Motif Chemokine Ligand 2
CCL5: C-C Motif Chemokine Ligand 5
COX1: Cyclooxygenase-1
COX2: Cyclooxygenase-2
CXCL8: C-X-C Motif Chemokine Ligand 8
EGFR: Epidermal Growth Factor Receptor
ERK: Extracellular Signal–Regulated Kinase
FDR: False Discovery Rate
HIF1: Hypoxia-Inducible Factor 1
IDO1: Indoleamine 2,3-Dioxygenase 1
IL6: Interleukin 6
IPA: Ingenuity Pathway Analysis
IRAK1: Interleukin 1 Receptor Associated Kinase 1
ITGAV: Integrin Subunit Alpha V
LDH: Lactate Dehydrogenase
MAPK: Mitogen Activated Protein Kinase
MCL1: Myeloid Cell Leukemia 1
MDSC: Myeloid Derived Suppressor Cells
MITF: Melanocyte Inducing Transcription Factor
mPGES1: Microsomal Prostaglandin E Synthase-1
mTOR: Mammalian Target Of Rapamycin
NFkB: Nuclear Factor kappa-light-chain-enhancer of activated B cells
PDL1: Programmed Death-Ligand 1
PGE2: Prostaglandin E2
PI3K: Phosphatidylinositol-4,5-Bisphosphate 3-Kinase
PTEN: Phosphatase and Tensin Homolog
PTGES: Prostaglandin E Synthase
PTGS2: Prostaglandin-Endoperoxide Synthase 2
qRT-PCR: Quantitative Real-Time Polymerase Chain Reaction
RCCS: Rotary Cell Culture System Bioreactor
ROCK1: Rho Associated Coiled-Coil Containing Protein Kinase 1
RPL13A: Ribosomal Protein L13a
siCOX2: Small interfering RNA COX2
STR: Short Tandem Repeat
sTRAIL: Soluble TNF-related apoptosis-inducing ligand
TLR4: Toll Like Receptor 4
TME: Tumor Microenvironment
TNFα: Tumor Necrosis Factor alpha
TRAF6: TNF Receptor Associated Factor 6
VEGFA: Vascular Endothelial Growth Factor A
