# Supplementary material for: miR-146a-5p impairs melanoma resistance to kinase inhibitors by targeting COX2 and regulating NFkB-mediated inflammatory mediators
Source: Cell Commun Signal. 2020 Sep 23;18:156. doi: 10.1186/s12964-020-00601-1 (PMC7510138; doi:10.1186/s12964-020-00601-1)
Supplement: Supplementary file 3 — Additional file 2: Supplementary Fig. S1. Altered miR-146a expression influences BRAF/MEKi sensitivity and apoptosis in melanoma. A) Boxplots representing IC50 values of 6 matched PLX4032-resistant (R) and sensitive (S) melanoma cell lines to PLX4032 (vemurafenib), BRAFi (dabrafenib), MEKi (trametinib), to the combined treatment BRAF/MEKi, and to sTRAIL-induced apoptosis. B) Inverse correlation between miR-146a expression levels and IC50 values of PLX4032, BRAFi and MEKi in melanoma cell lines (Spearman analysis). C) Forced expression of miR-146a (+m-miR-146a) in LM16R cell line and in LM69 and LM70 short term cultures increased the effects of PLX4032 treatment as shown by reduced cell growth and increased cell cytotoxicity and apoptosis, evaluated by CCK8, LDH and caspase 8 and 3/7 activity. D) Inhibition of miR-146a expression (i-miR-146a) in LM16 cells increased cell proliferation and decreased the release of LDH and the apoptosis rate as evaluated by CCK8, LDH and caspase 3/7 activity. E) Overexpression of miR-146a (m-miR-146a) upon PLX4032, BRAF/MEKi and sTRAIL treatments in LM47R cells increased cell cytotoxicity and apoptosis, as evaluated by LDH and caspase 8 and 3/7 activity. Data are plotted compared to scrambled control. *: p < 0.05, **: p < 0.01, ***: p < 0.0001 by Student’s unpaired t test. Supplementary Fig. S2. COX2 inhibition increases sensitivity to PLX4032 and to BRAF/MEKi and reduces PGE2 release. A) Relative luciferase activity after co-transfection of PTGS2 3’UTR luciferase reporter vector or control vector with miR-146a mimic or mimic negative control. Experiment was performed in LM16R cells. B) Inverse correlation between miR-146a expression levels and IC50 values of PLX4032 in melanoma cell lines (Spearman analysis). C) Western blot analyses showing downregulation of COX2 after transfection with siRNA against COX2 (siCOX2) compared to scrambled control (S). COX2 protein levels were downregulated to 15%, as determined by quantification of the [file 12964_2020_601_MOESM3_ESM.pdf]

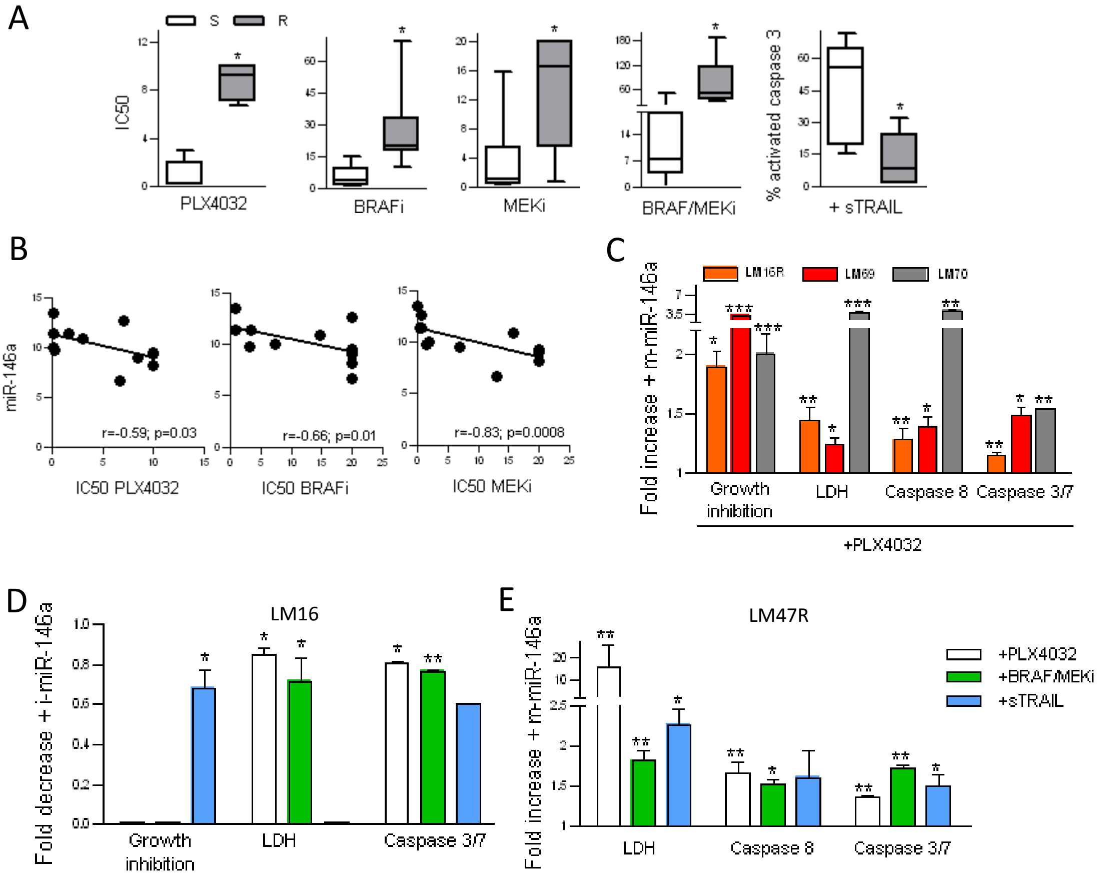

Supplementary figure S1

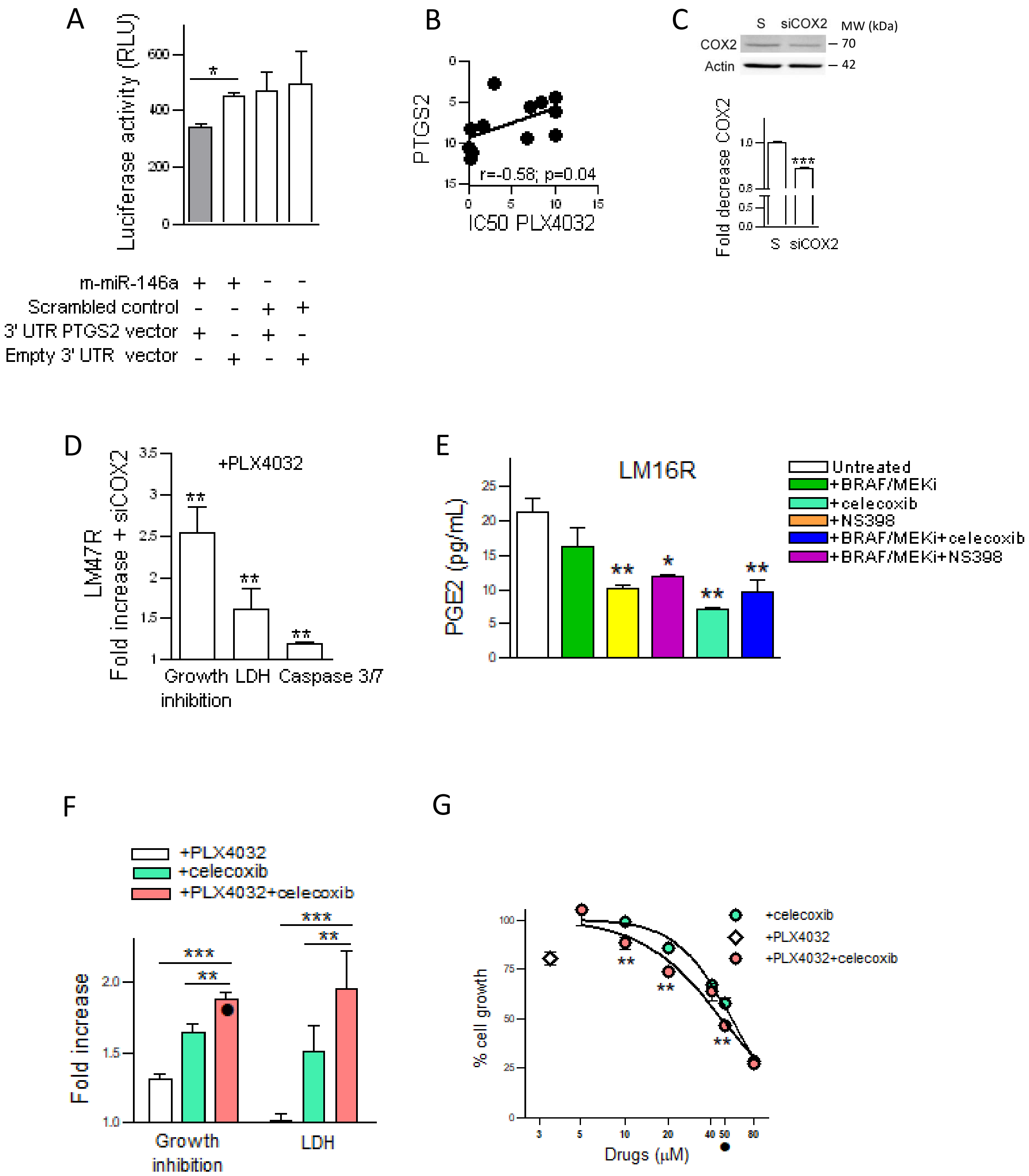

Supplementary figure S2

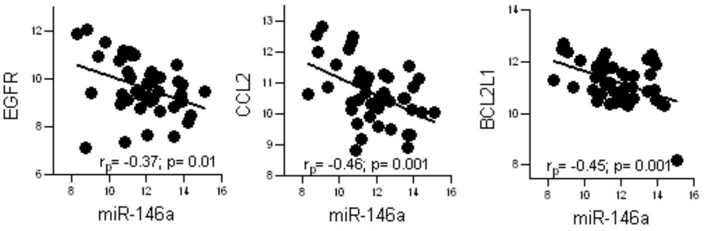

Supplementary figure S3

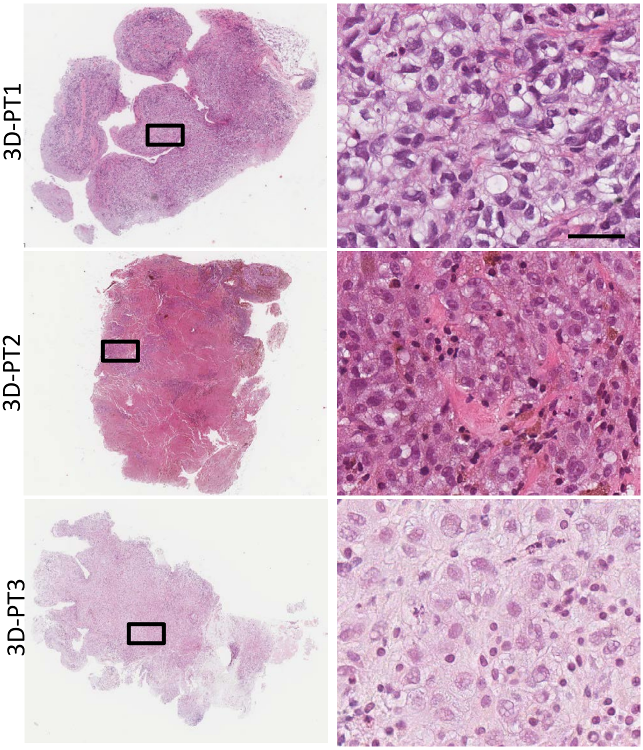

Supplementary figure S4

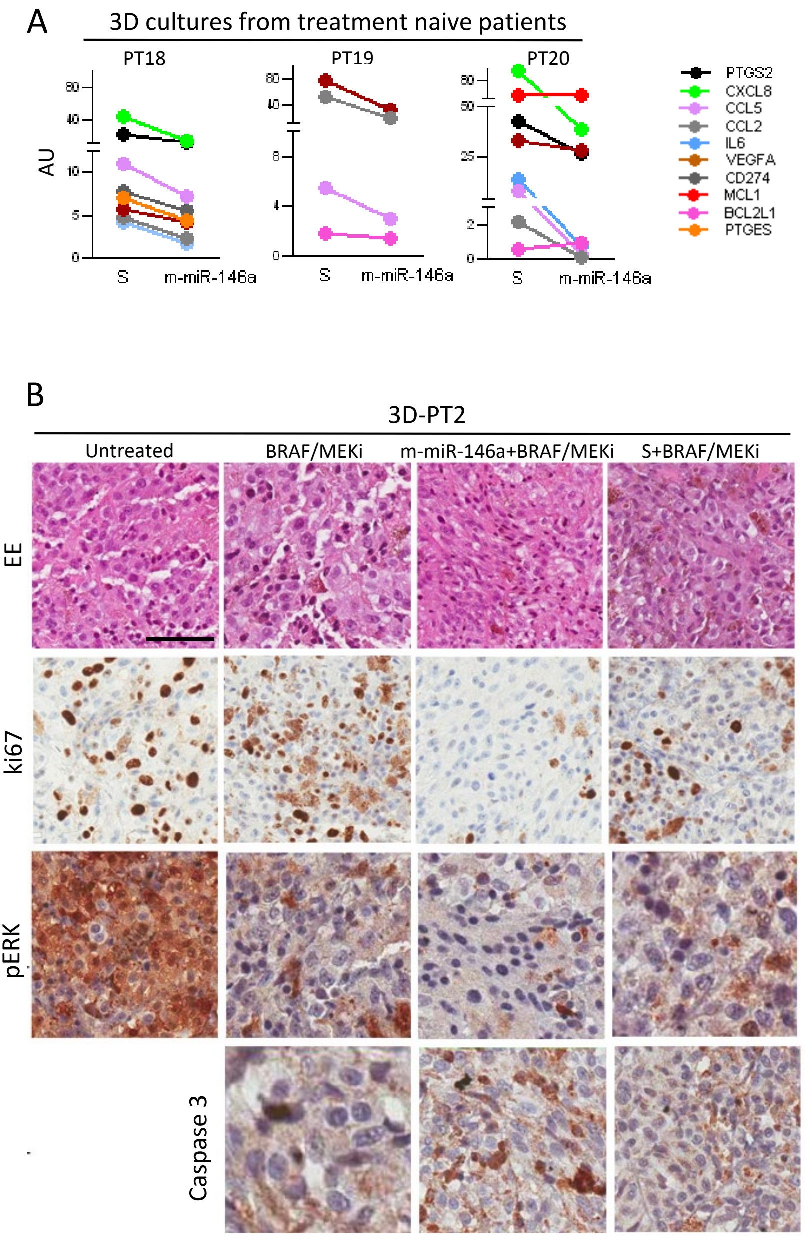

Supplementary figure S5
